# Can baseline ML Flow test results predict leprosy reactions? An investigation in a cohort of patients enrolled in the uniform multidrug therapy clinical trial for leprosy patients in Brazil

**DOI:** 10.1186/s40249-016-0203-0

**Published:** 2016-12-06

**Authors:** Emerith Mayra Hungria, Regiane Morillas Oliveira, Gerson Oliveira Penna, Lúcio Cartaxo Aderaldo, Maria Araci de Andrade Pontes, Rossilene Cruz, Heitor de Sá Gonçalves, Maria Lúcia Fernandes Penna, Ligia Regina Franco Sansigolo Kerr, Mariane Martins de Araújo Stefani, Samira Bührer-Sékula

**Affiliations:** 1 Tropical Pathology and Public Health Institute, Federal University of Goiás, Goiania, Goiás Brazil; 2 Tropical Medicine Centre, University of Brasília, Brasília, DF Brazil; 3 Dona Libânia Dermatology Centre, Fortaleza, Ceará Brazil; 4 Tropical Medicine Foundation/Foundation “Alfredo da Matta”, Manaus, Amazonas Brazil; 5 Epidemiology and Biostatistics Department, Federal University Fluminense, Niterói, Rio de Janeiro Brazil; 6 Department of Community Health, Federal University of Ceará, Fortaleza, Brazil

**Keywords:** Leprosy, Leprosy reactions, Bacillary index, Phenolic glycolipid-I, Clinical trial, ML Flow test, U-MDT/CT-BR, Brazil

## Abstract

**Background:**

The predictive value of the serology to detection of IgM against the *Mycobacterium leprae*-derived phenolic glycolipid-I/PGL-I to identify leprosy patients who are at higher risk of developing reactions remains controversial. Whether baseline results of the ML Flow test can predict leprosy reactions was investigated among a cohort of patients enrolled in The Clinical Trial for Uniform Multidrug Therapy for Leprosy Patients in Brazil (U-MDT/CT-BR).

**Methods:**

This was a descriptive study focusing on the main clinical manifestations of leprosy patients enrolled in the U-MDT/CT-BR from March 2007 to February 2012 at two Brazilian leprosy reference centers. For research purposes, 753 leprosy patients were categorized according to a modified Ridley-Jopling (R&J) classification and according to the development of leprosy reactions (reversal reaction/RR and erythema nodosum leprosum/ENL), and whether they had a positive or negative bacillary index/BI.

**Results:**

More than half of the patients (55.5 %) reported leprosy reaction: 18.3 % (138/753) had a RR and 5.4 % (41/753) had ENL. Leprosy reactions were more frequent in the first year following diagnosis, as seen in 27 % (205/753) of patients, while 19 % (142/753) developed reactions during subsequent follow-up. Similar frequencies of leprosy reactions and other clinical manifestations were observed in paucibacillary (PB) and multibacillary (MB) leprosy patients treated with U-MDT and regular MDT (R-MDT) (*P* = 0.43 and *P* = 0.61, respectively). Compared with PB patients, leprosy reactions were significantly more frequent in MB patients with a high BI, and more patients developed RR than ENL. However, RR and neuritis were also reported in patients with a negative BI. At baseline, the highest rate of ML Flow positivity was observed in patients with a positive BI, especially those who developed ENL, followed by patients who had neuritis and RR. Among reaction-free patients, 81.9 % were ML Flow positive, however, the differences were not statistically significant compared to reactional patients (*P* = 0.45).

**Conclusions:**

MB and PB patients treated with R-MDT and U-MDT showed similar frequencies of RR and other clinical manifestations. Positive ML Flow tests were associated with MB leprosy and BI positivity. However, ML Flow test results at baseline showed limited sensitivity and specificity for predicting the development of leprosy reactions.

**Electronic supplementary material:**

The online version of this article (doi:10.1186/s40249-016-0203-0) contains supplementary material, which is available to authorized users.

## Multilingual abstracts

Please see Additional file 1: for translations of the abstract into the six official working languages of the United Nations.

## Background

Leprosy, a complex chronic infectious disease caused by *Mycobacterium leprae*, primarily affects the skin and peripheral nerves and has a great potential to cause disability and irreversible deformities. Leprosy presents as a spectrum of manifestations: lepromatous leprosy (LL) is characterized by a high bacillary index, weak *M. leprae*-specific cell-mediated immunity (CMI), and high antibody titers. At the other extreme, tuberculoid (TT) patients have a low bacillary index, strong *M. leprae*-specific CMI, and weak antibody production. Immunologically unstable borderline tuberculoid (BT), borderline lepromatous (BL), and borderline-borderline (BB) forms lie in the middle of the spectrum combining features of both LL and TT forms [1].

During the chronic phase of leprosy, before diagnosis, during or after multidrug therapy (MDT), acute immune-inflammatory episodes, known as type 1 reactions or reversal reaction (RR) and type 2 reactions represented mainly by erythema nodosum leprosum (ENL), can lead to irreversible nerve damage [2, 3]. Moreover, neuritis characterized by intense spontaneous nerve pain is often associated with leprosy reactions, but can also occur without any cutaneous involvement [4]. Inappropriate therapeutic management of neuritis may result in permanent nerve function impairment [5, 6].

The World Health Organization (WHO) has been recommending MDT since 1981 [7]. Later, in 1988 a clinical classification based on the number of skin lesions was used to define two different MDT regimens [8] for either paucibacillary (PB) patients presenting up to five skin lesions or for multibacillary (MB) patients presenting more than five skin lesions. Multibacillary leprosy patients are prescribed 12 supervised monthly doses of rifampicin, dapsone, and clofazimine, plus self-administered daily doses of dapsone and clofazimine. Meanwhile, PB leprosy patients are treated with six supervised monthly doses of rifampicin and dapsone, plus self-administered daily doses of dapsone [7].

In 2007, an open-label, randomized clinical trial was conducted to compare regular MDT (R-MDT), as proposed by the WHO, and a uniform MDT (U-MDT) regimen consisting of six doses of rifampin, dapsone, and clofazimine for all leprosy patients (PB and MB) despite of their classification based on the number of lesions. The trial named the Clinical Trial for Uniform Multidrug Therapy Regimen for Leprosy Patients in Brazil (U-MDT/CT-BR) recruited leprosy patients in two Brazilian endemic sites which remain under clinical follow-up. Up until now, the total person-time observed is 780 930 person-days, i.e. 2 139.5 person-years, with a maximum of 6.66 years of follow-up [9].

Serology to detect immunoglobulin M (IgM) antibodies against the *M. leprae*-derived phenolic glycolipid-I (PGL-I) has been widely evaluated for leprosy classification, including its simple and rapid immunochromatographic ML Flow test which detects IgM to PGL-I in 10 min [10]. Studies have shown that the results of anti-PGL-I serology reflect the bacillary index (BI) of leprosy patients, with MB patients showing a high positivity rate (70 %–90 %) and PB patients showing low seropositivity (15 %–40 %) [11–13]. Conflicting data have been reported about the predictive value of anti-PGL-I serology for identifying patients who are at a higher risk of developing leprosy reactions. High levels of anti-PGL-I antibodies at diagnosis or after treatment have been associated with a higher risk of developing leprosy reactions [14–17], however, other studies have shown similar anti-PGL-I levels among reactional and reaction-free patients [18–20]. Therefore, whether anti PGL-I serology can be used to identify patients at risk of developing leprosy reactions remains unknown.

It is important to identify the risk factors for developing leprosy reactions in order to establish preventive strategies for reducing irreversible nerve damage and sequelae [21]. The present study describes the main clinical manifestations including leprosy reactions in a cohort of patients enrolled in the U-MDT/CT-BR. In addition, the baseline results of the ML Flow test for predicting the development of leprosy reactions observed during follow-up were evaluated.

## Methods

### Study population

This was a descriptive study focusing on the main clinical manifestations of leprosy patients enrolled in the U-MDT/CT-BR from March 2007 to February 2012 at two Brazilian leprosy reference centers: Dona Libânia (Fortaleza, Ceará state, northeast region) and Alfredo da Matta (Manaus, Amazonas state, north region), according to previously described rationale and study design [22].

Briefly, for the U-MDT/CT-BR study, following the WHO operational classification [8], patients were randomized into four groups: PB patients treated with U-MDT, PB patients treated with R-MDT, MB patients treated with U-MDT, and MB patients treated with R-MDT. In the R-MDT cohort, MB patients with six or more lesions were treated with 12 supervised monthly doses of rifampicin, dapsone, and clofazimine, plus daily self-administered doses of dapsone and clofazimine, while PB patients presenting five or fewer lesions received six supervised monthly doses of rifampicin and dapsone, plus daily self-administered doses of dapsone [23]. In the U-MDT cohort, six supervised monthly doses of rifampicin, dapsone, and clofazimine, plus daily self-administered doses of dapsone and clofazimine were prescribed for both PB and MB patients.

For research purposes, patients were also categorized according to a modified Ridley-Jopling (R&J) classification system taking into account clinical features, histopathological results of skin biopsies, and the BI on slit skin smear; Mitsuda tests and BI of the skin biopsy were not performed. This modified R&J classification taking into account the development of reactions was used to write this paper.

Our study group included 753 out of 859 patients enrolled in the U-MDT/CT-BR; 12.3 % (106/859) of patients were excluded due to: incomplete treatment (*n* = 9), histopathological diagnosis not compatible with leprosy (*n* = 3), patients requested to leave the study (*n* = 8), death (*n* = 19), transfer of patients to other sites (*n* = 9), patients lost to follow-up (*n* = 12), adverse drug reactions (*n* = 25), other concomitant illnesses (*n* = 5), leprosy relapse (*n* = 1), or other reasons (*n* = 15). The patients’ clinical information was obtained from the case report form, at baseline, and during follow-up (from March 2007 to September 2013). This included a monthly medical visit in the first year and a yearly medical visit in the following 6 years; clinical monitoring is still taking place. As part of protocol, at enrollment, all patients were advised about the signs and symptoms of leprosy reactions and nerve function impairments, and advised to immediately return to the clinic in case any abnormality occurred.

### Case definitions for leprosy reactions and neuritis

A RR was defined as an acute inflammation of pre-existing cutaneous lesions with or without the appearance of new lesions. The clinical diagnosis of ENL was based on the acute appearance of erythematous nodular skin lesions, tender to touch or painful in the absence of external stimuli, accompanied by fever with or without peripheral nerve pain and dysfunction. Neuritis was defined as spontaneous nerve pain or by the presence of thickened nerve trunks associated with loss of sensation and motor and autonomic deficits. Study groups included reaction-free patients, patients developing a RR, patients with ENL, and a group of patients with unusual clinical manifestations such as necrotizing ENL, mixed RR-ENL reactions, polymorphic erythema, arthritis, lymphadenopathy, orchitis, iritis, iridocyclitis, and reactional hands and feet. Dermatologists with vast expertise in leprosy diagnosis, treatment, and patients’ management were in charge of the clinical diagnosis and follow-up of leprosy reactions. Patients who developed leprosy reactions or who had impaired nerve function received appropriate treatment according to the guidelines of the National Leprosy Control Program/Brazilian Ministry of Health and remained in the study; RR, ENL, and associated clinical manifestations were registered in the case report form (CRF).

### ML Flow serological tests

The ML Flow test was performed, as previously described elsewhere [10]. Briefly, 5 μl of whole blood and running buffer were added to the sample well and visual readings were performed 10 min later. Results were recorded according to the scale of positivity: 0 and 0.5 = negative; 1 +, 2 +, 3 +, and 4 + = positive [10].

### Statistical analyses

Leprosy patients were stratified into subgroups, as either patients who had a reactional episode or reaction-free patients. Episodes of RR and ENL were analyzed individually or associated with neuritis. Statistical significance was assessed using the chi-square (*χ*2) test to compare the reactional and reaction-free groups. Results were considered statistically significant when *P*-values were <0.05. Descriptive analyses of the clinical, epidemiological, and laboratory variables and box plots were performed using SPSS version 21 (IBM, New York, USA). Receiver operating characteristic (ROC) curves were determined with 95 % confidence intervals (CIs) (using GraphPad Prism Software version 5, CA, USA).

### Ethical considerations

The UMDT/CT-BR was performed under international (Helsinki) and Brazilian research regulations regarding human beings and was approved by regional ethics committees from all states involved in the study, as well as by the National Commission for Ethics in Research of the National Health Council/Ministry of Health (February 17, 2006, protocol number 001/06). Written informed consent was obtained from all patients prior to their inclusion in the study. For patients aged below 18 years, written parental consent was obtained. Data confidentiality was strictly guaranteed and all patients knew they were free to leave the study and opt for the R-MDT regimen outside of the study at any time (ClinicalTrials.gov identifier: NCT00669643).

## Results

### U-MDT/CT-BR: Clinical and laboratory features of patients at baseline and during follow-up

Of the 753 patients enrolled in the U-MDT/CT-BR, 80.5 % (606/753) were from Ceará and 19.5 % (147/753) were from Amazonas. In terms of classification, 21 % (158/753) were PB patients and 79.0 % (595/753) were MB patients. The majority of PB patients were female and reaction-free PB patients had the lowest median age in PB group (34 years) (see Table 1). Most MB patients were male, and MB patients who developed ENL had the lowest median age in MB group (33 years).

**Table 1:** Main characteristics of the 753 leprosy patients from UMDT/CT-BR study in PB and MB cases stratified according to the development of leprosy reaction and other manifestations

	Number	Sex (F/M)	Age (median, range)	R&J	BI (median,range)	BCG scar (presence of scar/total)
PB reaction-free	129	86/43	34 (9-65)	11 I/ 42TT/ 75 BT/ 1BB	0 (0-1.75)	66/129
PB with RR	13	8/5	38 (6-60)	3 TT/ 7 BT/ 3 BB	0 (0-2.6)	6/13
PB with neuritis	16	10/6	39 (11-63)	3 TT/ 12BT/1BB	0 (0-0.75)	7/16
MB reaction-free	206	91/115	43 (8-65)	115 BT/ 9 BB/ 44 BL/ 38 LL	0 (0-6)	106/206
MB with RR	153	35/118	43 (7-65)	30 BT/ 17 BB/106 BL	2.75 (0-6)	78/153
MB with ENL	48	16/32	33 (8-62)	15 BL/ 33 LL	4.5 (0.5-5.6)	23/48
MB with others CM	55	17/38	45 (11-65)	9 BT/ 3 BB/ 26 BL/ 17 LL	4 (0-6)	30/55
MB with neuritis	133	42/91	36 (10-64)	47 BT/ 7 BB/ 43 BL/ 36 LL	4 (0-5.75)	53/133

*PB* Paucibacillary Leprosy, *MB* Multibacillary Leprosy, *RR* Reversal Reaction, *ENL* Erythema Nodosum Leprosum, *CM* clinical manifestations, *n* number, *F* female, *M* male, *R & J* Ridley & Jopling Classification, *BI* Bacilloscopic Index, *BCG* Bacillus Calmette Guérin vaccine. PB and MB leprosy cases classification was based on the number of skin lesions according to WHO operational classification system

In this cohort, 55.5 % (418/753) of patients had at least one episode of a leprosy reaction during follow-up. The distribution of the first leprosy episode according to R&J groups is presented in Table 2. The most significant portion of reactional patients had either BT or BL leprosy (39 %; 294/753 and 31.1 %; 234/753, respectively). This was followed by LL (16.5 %; 124/753), TT (6.4 %; 48/753), and BB (5.6 %; 42/753). Only 1.5 % (11/753) had indeterminate leprosy. The most prevalent type of reaction was the RR, affecting 18.3 % (138/753) of patients and occurring mainly in borderline patients (BT, BB, BL) (136/138). Meanwhile, ENL was observed in 5.4 % (41/753) of patients (all who had BL or LL). Neuritis alone was observed in 19.8 % (149/753) of patients. Among the patients who had a reaction, 21.5 % (90/418) had reactions associated with other clinical manifestations, such as neuritis, orchitis, arthritis, and lymphadenopathy. The majority of neuritis cases (59/149) and reaction-free patients (190/335) had BT leprosy.

**Table 2:** Distribution of first episode of leprosy reactions and main clinical manifestations developed by patients stratified by R&J groups

	I	TT	BT	BB	BL	LL	TOTAL (%)
Reactions	0	6	105	31	190	86	418 (55.5)
RR	0	2	28	16	92	0	138 (18.3)
ENL	0	0	0	0	13	28	41 (5.4)
Necrotising ENL	0	0	0	0	0	1	1 (0.1)
Lymphadenopathy	0	0	0	0	1	1	2 (0.2)
Reaction hand and foot	0	0	4	1	8	6	19 (2.5)
ENL + Polymorphic Erythema	0	0	0	0	1	0	1 (0.1)
ENL + Orchitis	0	0	0	0	0	1	1 (0.1)
ENL + Arthritis	0	0	0	0	0	1	1 (0.1)
ENL + Lymphadenopathy	0	0	0	0	1	1	2 (0.3)
ENL + reactional hand and foot	0	0	0	0	0	3	3 (0.4)
Mixed reaction / Type 1 + Type 2	0	0	0	1	6	1	8 (1.1)
Neuritis	0	3	59	8	43	36	149 (19.8)
Neuritis + RR	0	1	9	4	14	0	28 (3.7)
Neuritis + ENL	0	0	0	0	2	5	7 (0.9)
Neuritis + Mixed reaction	0	0	0	0	1	0	1 (0.1)
Neuritis + Arthritis	0	0	1	0	1	0	2 (0.3)
Neuritis + Orchitis	0	0	1	0	0	0	1 (0.1)
Neuritis + reactional hand and foot	0	0	3	1	7	1	12 (1.6)
Neuritis + ENL + polymorphic erythema	0	0	0	0	0	1	1 (0.1)
Reaction-free	11	42	190	10	44	38	335 (44.5)
Total	11	48	295	41	234	124	753 (100)

The distribution of reactions according to the WHO operational classification is presented in Table 3. Leprosy reactions were significantly more frequent in the MB group (93.1 %; 389/418) compared with the PB group (6.9 %; 29/418) (*P* < 0.001). Among MB patients, a significant proportion had a RR (21.5 %; 128/595), while 6.9 % (41/595) had ENL. Almost one-third of the MB patients (27.7 %; 165/595) had neuritis alone or neuritis associated with RR or ENL, and 9.2 % (55/595) presented other unusual clinical manifestations such as lymphadenopathy or polymorphic erythema. Out of the PB cases who had a reaction, 6.3 % (10/158) developed a RR and 12 % (19/158) had neuritis alone or neuritis associated with RR.

**Table 3:** Reactional status of UMDT/CT-BR leprosy patients according to operational classification

	PB N (%)	MB N (%)	Total N (%)
Reactions	29 (3.9)	389 (51.6)	418 (55.5)
RR	10 (1.3)	128 (17)	138 (18.3)
ENL	0	41 (5.4)	41 (5.4)
RR + Neu	3 (0.4)	25 (3.3)	28 (3.7)
ENL + Neu	0	7 (0.9)	7 (0.9)
Neuritis	16 (2.1)	133 (17.7)	149 (19.8)
Others	0	55 (7.3)	55 (7.3)
Reaction-free	129 (17.1)	206 (27.4)	335 (44.5)
Total	158 (21)	595 (79)	753 (100)

*X* ^2^_(6)_ = 118.97; *p* < 0.001

*PB* Paucibacillary Leprosy, *MB* Multibacillary Leprosy, *RR* Reversal Reaction, *ENL* Erythema Nodosum Leprosum, *Neu* Neuritis, *Others* Other Manifestations

In this study group, 56.6 % (426/753) of patients were positive for slit skin smears (mean BI = 3.39; SD = 1.49). Patients with a positive BI smear (75.4 %; 321/426) were more likely to develop a leprosy reaction as compared with patients with a negative BI smear (29.7 %; 97/327) (*P* < 0.001) (see Table 4). Out of the reactional patients with a positive BI, 34.9 % (112/321) had a RR, 29.3 % (94/321) had neuritis, and 12.8 % (41/321) developed ENL. Almost half of the patients with a positive BI (46.5 %; 198/426) developed leprosy reactions in the first year following diagnosis.

**Table 4:** Leprosy-reactional status of 753 patients enrolled at UMDT/CT-BR according to the bacterial index

	BI Negative N (%)	BI Positive N (%)	Total N (%)
Reactions	97 (12.9)	321 (42.6)	418 (55.5)
RR	26 (3.4)	112 (14.9)	138 (18.3)
ENL	0	41 (5.4)	41 (5.4)
RR + Neu	9 (1.2)	19 (2.5)	28 (3.7)
ENL + Neu	0	7 (0.9)	7 (0.9)
Neuritis	55 (7.3)	94 (12.5)	149 (19.8)
Others	7 (0.9)	48 (6.4)	55 (7.3)
Reaction-free	230 (30.5)	105 (14)	335 (44.5)
Total	327 (43.4)	426 (56.6)	753 (100)

*X* ^2^_(6)_ = 182.7; *p* < 0.001

*BI* Bacilloscopic Index, *RR* Reversal reaction, *ENL* Eritema Nodosum Leprosum, *Neu* neuritis, *Others* others manifestations

### Time of first episode of leprosy reaction

In this cohort, around 10 % patients had a leprosy reaction at diagnosis (9.4 %, 71/753): 22.5 % (16/71) of these patients had RR and 4.2 % (3/71) had ENL plus neuritis (see Table 5). Thereafter, leprosy reactions were more frequent in the first year after diagnosis, affecting 27.2 % (205/753) of patients (RR: 34.6 %, 71/205; ENL: 4.4 %, 9/205; neuritis: 37.6 %, 77/205), while 19 % (142/753) of patients developed reactions during subsequent follow-up (RR: 35.9 %, 51/142; ENL: 22.5 %, 32/142). The development of ENL was more frequent in the first 12 months after treatment compared to further follow-up (*P* = 0.02). Overall, the development of leprosy reactions or other clinical manifestations was more frequent in the first year following diagnosis (27.2 %, 205/753 versus 19 %, 142/753; *P* = 0.003).

**Table 5:** Time of presentation of leprosy reaction among UMDT/CT-BR patients according to type of reaction

	At diagnosis N (%)	In the 1^st^ year after diagnosis N (%)	Follow-up after 1^st^ year N (%)	Reaction-free N (%)	Total N (%)
RR	16 (22.5)	71 (34.6)	51 (35.9)	NA	138 (18.3)
ENL	0	9 (4.4)	32 (22.5)	NA	41 (5.4)
RR + Neu	6 (8.5)	15 (7.3)	7 (4.9)	NA	28 (3.7)
ENL + Neu	3 (4.2)	3 (1.5)	1 (0.7)	NA	7 (0.9)
Neuritis	43 (60.6)	77 (37.6)	29 (20.4)	NA	149 (19.8)
Others	3 (4.2)	30 (14.6)	22 (15.5)	NA	55 (7.3)
Total	71 (100)	205 (100)	142 (100)	335 (100)	753 (100)

*RR* Reversal reaction, *ENL* Eritema Nodosum Leprosum, *Neu* neuritis, *Others* others manifestations

A similar frequency of leprosy reactions and other clinical manifestations was observed among PB patients being treated with U-MDT compared with PB patients being treated with R-MDT (22.1 %, 17/77 versus 14.6 %, 12/82; *P* = 0.43). In addition, PB patients from both treatment groups developed a RR (6/77 in the U-MDT group and 4/82 in the R-MDT group). A similar frequency of leprosy reactions was also seen among MB patients being treated with U-MDT compared with MB patients being treated with R-MDT (66.7 %, 214/321 versus 64.1 %, 175/273; *P* = 0.61) (see Table 6).

**Table 6:** Leprosy reactions among patients enrolled at UMDT/CT-BR according to treatment group

	PB UMDT N (%)	PB RMDT N (%)	MB UMDT N (%)	MB RMDT N (%)	Total N (%)
Reactions	17 (22.1)	12 (14.6)	214 (66.7)	175 (64.1)	418 (55.5)
RR	6 (7.8)	4 (4.9)	69 (21.5)	59 (21.6)	138 (18.3)
ENL	0	0	20 (6.2)	21 (7.7)	41 (5.4)
RR + Neu	1 (1.3)	2 (2.4)	15 (4.7)	10 (3.7)	28 (3.7)
ENL + Neu	0	0	5 (1.6)	2 (0.7)	7 (0.9)
Neuritis	10 (13)	6 (7.3)	79 (24.6)	54 (19.8)	149 (19.8)
Others	0	0	26 (8.1)	29 (10.6)	55 (7.3)
Reaction-free	60 (77.9)	70 (85.4)	107 (33.3)	98 (35.9)	335 (44.5)
Total	77 (100)	82 (100)	321 (100)	273 (100)	753 (100)

For the statistical analysis of PB patients were excluded ENL and ENL + neuritis. RR and RR + neuritis were grouped

*RR* Reversal reaction, *ENL* Eritema Nodosum Leprosum, *Neu* neuritis, *Others* others manifestations

PB – *x* ^2^_(2)_ = 1.69; *p* = 0.43; MB – *x* ^2^_(6)_ = 0.61; *p* = 0.61

### ML Flow test results at baseline

**Fig. 1:**
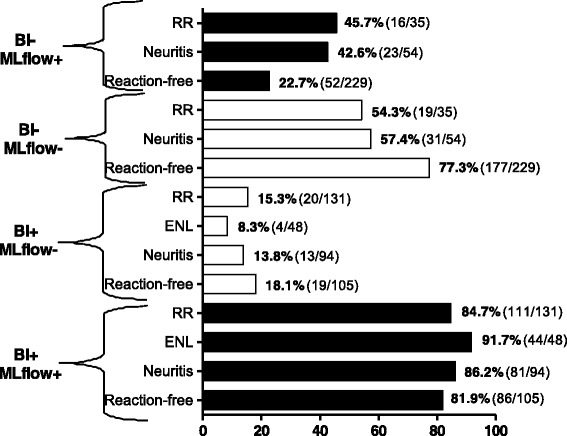
ML Flow test results stratified according to BI and reaction type. Legend: ML Flow result was missing for one patient

At baseline, higher seropositivity in the ML Flow was recorded among patients with a positive BI as compared with patients with a negative BI (86 %; 366/426 versus 29.1 %; 95/327) (*P* <0.001). The baseline serologic profile of patients evaluated by the ML Flow test showed higher positivity in patients who developed reactions (77 %; 322/418) as compared with reaction-free patients (41.2 %; 138/335) (*P* <0.001) (see Fig. 1) (see also Additional file 2: Table S1).

**Fig. 2:**
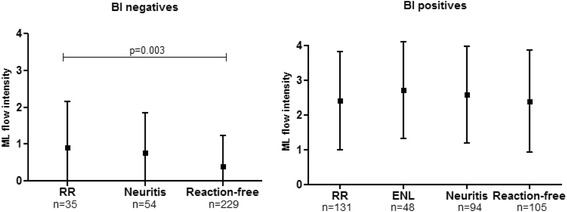
Intensity of positive ML Flow tests, according to BI and type of reaction

At baseline, the highest rate of ML Flow positivity and the highest colour intensity of readings were recorded, as expected, among patients with a positive BI from the following categories: patients who developed ENL or ENL associated with neuritis (92 %, 44/48; mean ML Flow reading score: 2.7), followed by patients who presented with neuritis (86.2 %, 81/94; mean ML Flow reading score: 2.6) and patients who developed a RR (84.7 %, 111/131; mean ML Flow reading score: 2.4). Reaction-free patients had the lowest positivity (81.9 %, 86/105; mean ML Flow reading score: 2.4), however, differences among the groups were not statistically significant (*P* = 0.45). Among the patients with a negative BI, those who developed a RR or neuritis had a higher ML Flow positivity rate and higher colour intensity in test readings compared with reaction-free patients (RR: 45.7 %, 16/35, mean ML Flow reading score: 0.9; neuritis: 42.6 %, 23/54, mean ML Flow reading score: 0.8; reaction-free: 22.7 %, 52/229, mean ML Flow reading score: 0.4) (*P* < 0.001) (see Figs. 1 and 2). Among the patients who had a positive BI, the strength of the ML Flow signal was similar in patients who had a reaction (ENL, RR, or neuritis) and reaction-free patients (*P* > 0.05), while among the patients who had a negative BI, a higher signal strength was observed in patients who developed RR as compared with the reaction-free patients (*P* = 0.003) (see Fig. 2).

The accuracy of the ML Flow test at baseline to predict whether a patient will develop leprosy reactions was analyzed using the ROC curve. For patients who had a negative BI and developed a RR during follow-up or who did not have a reaction, the area under the curve was <0.7. Establishing a specificity of 90 % (95 % *CI*: 85–93 %), the sensitivity was 28 % (95 % *CI*: 14–46 %) for a reading score of ≥2. Similar results were observed in patients who had a positive BI and developed ENL or RR (see Additional file 3: Figure S1).

## Discussion

For many years, leprosy patients were treated with dapsone monotherapy, and the development of ENL was reported in half of LL patients and in one-quarter of BL patients [24]. The introduction of MDT in the 1980s reduced the frequency and the severity of ENL, probably as a result of the anti-inflammatory activity of clofazimine that was included in the treatment regimen [25]. However, previous data from the U-MDT cohort showed that there was no statistical difference in the frequency of leprosy reactions among MB patients receiving R-MDT or U-MDT, indicating that six or 12 months of clofazimine treatment does not result in the prevention of leprosy reactions [26]. In the U-MDT/CT-BR, until 2014, 780 930 person-days, i.e. 2 139.5 person-years, with a maximum of 6.66 years of follow-up were evaluated [9]. In the current study, a comparable frequency of leprosy reactions and other clinical manifestations was observed in PB patients being treated with U-MDT or R-MDT. Findings from a previous report together with findings from the current investigation indicate that whether patients receive U-MDT or R-MDT does not have any impact on the incidence of leprosy reactions and other clinical manifestations such as neuritis.

In this cohort of leprosy patients, composed mainly of MB patients (~80 %), more than half developed at least one episode of a leprosy reaction. Moreover, the great majority of patients who developed reactions were MB patients with high BIs, and these variables have been previously associated with a high probability of developing leprosy reactions [2, 27, 28]. Our results confirm that MB patients have a higher risk of developing leprosy reactions as compared with PB patients. In addition, the study confirms that the most common reaction in both MB and PB patients is RR, and ENL occurs only in MB patients. While most MB patients developed reactions, the majority of PB patients remained reaction-free. Overall, more than half of the patients had a positive BI and reactions were more frequent in these patients as compared with patients who had a negative BI. The development of reactions was more common in the first year of follow-up compared to subsequent years. Our results are in accordance with other studies, which showed the predominance of RR over ENL and the higher incidence of reactions in MB patients with a positive BI as compared with patients with a negative BI [29, 30].

In this cohort, RRs were seen mainly in BL MB patients, a finding which is consistent with other studies [2, 14, 31]. The majority of patients who developed ENL had LL and high BIs. Other studies have reported that ENL is more often seen in LL patients than in BL patients [32, 33] and a higher BI is a known risk factor for developing ENL [34, 35]. Interestingly, MB patients who developed ENL were younger than reaction-free MB patients. Accordingly, a study that evaluated risk factors for developing ENL showed that patients older than 40 years were less likely to develop ENL [34]. Another study showed that patients whose first leprosy symptom occurred during adolescence had a greater chance of developing ENL than patients whose onset of leprosy occurred after adolescence [36]. In this study, PB patients who developed RR were older than reaction free-PB patients. Other studies have shown that older age was an important risk factor for developing both RR at diagnosis and developing sequelae after treatment [37, 38].

During MDT, bacillary death occurs resulting in a massive release of mycobacterial antigens favoring the formation of immune complexes, mainly among MB patients, and immune complexes are considered to play a role in the pathophysiology of leprosy reactions [39–42]. In this study, reactions were more frequent during the course of MDT, as previously reported [2, 27, 43]. However, patients mainly developed ENL during follow-up. The results of this study underline the importance of alerting patients about the possible development of reactions before, during, and even years after the conclusion of MDT, since reactions require immediate assistance and specific treatment to avoid irreversible nerve damage. Overall, neuritis was a common clinical manifestation that occurred in all clinical forms of leprosy, with higher frequencies observed in BT and BL forms. Similar to previously reported data, isolated neuritis with no other dermatological or clinical symptom was present in 20 % of cases, while less than half of the neuritis cases were associated with a reaction [44].

In this study, baseline positivity determined using the ML Flow test was associated with MB disease, a positive BI, and the development of reactions during follow-up, mainly ENL. Bacillary load is known to directly correlate with antibody levels and with the development of leprosy reactions [2, 16, 27, 28]. Moreover, antibodies, which are abundant in MB patients, probably play a role in the pathophysiology of ENL [15–17, 39]. However, little, if anything, is known about the role of antibodies in the development of RRs and other clinical manifestations such as neuritis. In this study, at baseline, patients with a negative BI who developed neuritis and RRs during follow-up had higher ML Flow positivity with a higher colour intensity compared with reaction-free patients who had a negative BI. Further studies on the immunopathogenesis of RRs are needed in order to clarify whether antibodies play a role in the development of RRs.

Our ROC analysis showed that the results of the ML Flow test at baseline had limited sensitivity and specificity to predict whether patients will develop leprosy reactions during follow-up (see Additional file 3: Figure S1). Additionally, high ML Flow seropositivity was not always associated with leprosy reactions and other clinical manifestations, as high positivity was also observed in reaction-free patients.

## Conclusions

In the U-MDT/CT-BR, until 2014, 780 930 person-days, i.e. 2 139.5 person-years, with a maximum of 6.66 years of follow-up were evaluated, in this period MB and PB patients receiving R-MDT or U-MDT showed similar frequencies of RR, ENL and other clinical manifestations. Moreover, our data confirm that MB patients with a positive BI are more likely to develop leprosy reactions, and overall RRs are more common than ENL. Leprosy reactions were more likely to happen in the first year post-MDT, suggesting that special emphasis needs to be placed on clinical monitoring during this period. A significant proportion of patients presented neuritis alone or neuritis associated with reactions. ML Flow test results at baseline showed limited sensitivity and specificity for predicting whether patients will develop leprosy reactions during follow-up. Positive ML Flow tests were associated with MB disease, having a positive BI, and developing reactions, particularly ENL. As well as that, RR and neuritis were also reported in patients with a negative BI, and these patients had more positive ML Flow test results compared with the reaction-free patients who had a negative BI. Therefore ML Flow test results at baseline cannot be used as a predictive marker for leprosy reactions. Further studies to investigate other quantitative serological markers, besides anti PGL-I response, may help us to further understand the role of serology as a predictive tool for leprosy reactions.

## Additional files

Additional file 1: Multilingual abstracts in the six official working languages of the United Nations. (PDF 697 kb)
Additional file 2: Table S1. Positivity to ML Flow test according to bacillary index (BI) and type of reaction. (DOC 43 kb)
Additional file 3: Figure S1. Receiver Operating Curves (ROC) for ML Flow test in leprosy patients who developed RR, ENL and reaction-free. (DOC 715 kb)

## Abbreviations

BB: Borderline-borderline
BI: Bacillary index
BL: Borderline lepromatous
BT: Borderline tuberculoid
CI: Confidence interval
CMI: Cell-mediated immunity
ENL: Erythema nodosum leprosum
LL: Lepromatous leprosy
MB: Multibacillary
MDT: Multidrug therapy
PB: Paucibacillary
PGL-I: Phenolic glycolipid-I
R&J: Ridley-Jopling
R-MDT: Regular multidrug therapy
ROC: Receiver operating characteristic
RR: Reversal reaction
TT: Tuberculoid
U-MDT: Uniform multidrug therapy
U-MDT/CT-BR: Clinical trial for uniform multidrug therapy for leprosy patients in Brazil
WHO: World Health Organization
