# Supplementary material for: Can baseline ML Flow test results predict leprosy reactions? An investigation in a cohort of patients enrolled in the uniform multidrug therapy clinical trial for leprosy patients in Brazil
Source: Infect Dis Poverty. 2016 Dec 6;5:110. doi: 10.1186/s40249-016-0203-0 (PMC5139020; doi:10.1186/s40249-016-0203-0)
Supplement: Additional file 1: — Multilingual abstracts in the six official working languages of the United Nations. (PDF 697 kb) [file 40249_2016_203_MOESM1_ESM.pdf]

Translation of the abstract into the five official working languages of the United Nations

هل يمكن لنتائج اختبار تدفق خط الأساس للمتفطرة الجذامية أن تتوقع ردود فعل الجذام؟ بحث في مجموعة من المرضى المسجلين في تجربة سريرية لعلاج متعدد الدواء موحد لمرضى الجذام في البرازيل

إيميريث مايرا هنجيريا، ريجيان موريلاس أوليفيرا، جيرسون أوليفيرا بينا، لوسيو كارتاكسو أديرالدو، ماريا آراكي دي أندرادي بونتيس، روزيلين كروز، إيتور دي سا جونسالفيس، ماريا لوسيا فرنانديز بينا، ليجيا ريجينا فرانكو سانسيجولو كير، ماريان مارتينز دي أروجو ستيفاني، سميرا بيرر- سيكولا

الملخص

الخلفية: القيمة التنبؤية للأمصال في الكشف عن الجلوبولين المناعي ضد الشحمي السكري الفينولي I / PGL-I- المشتق من المتفطرة-الجذامية لتحديد مرضى الجذام الذين هم أكثر عرضة لنمو ردود أفعال لا زالت مثيرة للجدل. وإذا ما كانت نتائج خط الأساس لاختبار تدفق المتفطرة الجذامية يمكنها التنبؤ بردود فعل الجذام تم بحثها بين لفيف من المرضى المسجلين في التجربة السريرية لعلاج دوائي متعدد موحد لمرضى الجذام في البرازيل (U-MDT / CT-BR).

الطرق: كانت هذه دراسة وصفية تركز على المظاهر السريرية الرئيسية لمرضى الجذام المسجلين في U-MDT / CT-BR من مارس 2007 إلى فبراير 2012 في مركزي مراجعة لمرضى الجذام بالبرازيل. لأغراض البحث، تم تصنيف 753 مريض بالجذام وفقاً لتصنيف ريدلي-جوبلينج (R&J) المعدّل ووفقاً لتطور ردود فعل الجذام (رد فعل إنعكاسي / RR وحمامي عقدية ورمية / ENL)، وعما إذا كان لديهم مؤشر عصوي/ BI إيجابي أو سلبي.

النتائج: أبلغ أكثر من نصف المرضى (55.5٪) عن رد فعل الجذام: 18.3٪ (753/138) كان لديهم رد فعل إنعكاسي و 5.4٪ (753/41) لديهم حمامي عقدية ورمية. وكانت ردود فعل الجذام أكثر تواتراً في السنة الأولى بعد التشخيص، كما شوهد في 27٪ (753/205) من المرضى، في حين ظهرت ردود الفعل لـ 19٪ (753/142) خلال متابعة لاحقة. وقد لوحظت ترددات مماثلة من ردود فعل الجذام ومظاهر سريرية أخرى في مرضى الجذام قليل العصيات (PB) ومتعدد العصيات (MB) المعالجين بـ U-MDT و (R-MDT) العادي (P = 0.43 و P = 0.61، على التوالي). مقارنة مع مرضى الجذام قليل العصيات، كانت ردود فعل الجذام أكثر تواتراً بشكل ملحوظ في مرضى الجذام متعدد العصيات مع مؤشر عصوي عالٍ، وظهر للمزيد من المرضى رد فعل إنعكاسي بالمقارنة مع الحمامي العقدية الورمية. ومع ذلك، تم الإبلاغ عن رد فعل إنعكاسي والتهاب العصب أيضاً في المرضى الذين لديهم مؤشر عصوي سلبي. في الأساس، لوحظ أن أعلى معدل لإيجابية تدفق المتفطرة الجذامية كان في المرضى الذين لديهم مؤشر عصوي إيجابي، وخاصة أولئك الذين ظهر لديهم حمامي عقدية ورمية، يليهم المرضى الذين لديهم التهاب العصب ورد فعل إنعكاسي. بين المرضى الخالين من رد فعل، كان لدى 81.9٪ تدفق المتفطرة الجذامية إيجابي، ومع ذلك، كانت الفروق غير هامة إحصائياً بالمقارنة مع المرضى ذوي ردود الأفعال (P = 0.45).

الاستنتاجات: ظهر لمرضى الجذام متعدد العصيات وقليل العصيات المعالجين بـ R-MDT وU-MDT ترددات مماثلة من رد فعل إنعكاسي ومظاهر سريرية أخرى. وارتبطت إيجابية اختبارات تدفق المتفطرة الجذامية مع مرضى الجذام متعدد العصيات وإيجابية المؤشر العصوي. ومع ذلك، أظهرت نتائج اختبار تدفق المتفطرة الجذامية في الأساس حساسية محدودة وخصوصية للتنبؤ بتطور تفاعلات الجذام.

Translated from English version into Arabic by free bird, through

TRANSLATORS
WITHOUT BORDERS

基线 ML 明窗实验（ML Flow test）结果能否预测麻风反应？
——巴西关于统一多药治疗麻风患者临床试验的队列研究

Emerith Mayra Hungria, Regiane Morillas Oliveira, Gerson Oliveira Penna, Lúcio Cartaxo Aderaldo, Maria Araci de Andrade Pontes, Rossilene Cruz, Heitor de Sá Gonçalves, Maria Lúcia Fernandes Penna, Ligia Regina Franco Sansigolo Kerr, Mariane Martins de Araújo Stefani, Samira Bührer-Sékula

摘要

引言：检测麻风患者抗麻风分枝杆菌苯酚糖脂 I（PGL-I）IgM 抗体水平，以确定哪些患者具有更高风险的方法仍存在争议。那么，基线 ML 明窗实验（ML Flow test）结果是否能预测麻风反应，在巴西开展了一项关于统一多药治疗麻风患者临床试验（Uniform Multidrug Therapy for Leprosy Patients in Brazil，U-MDT/CT-BR）的队列研究。

方法：该研究对巴西两个麻风病参考中心 2007 年 3 月至 2012 年 2 月纳入 U-MDT/CT-BR 的麻风患者的主要临床表现进行描述。根据改良的 Ridley-Jopling 分类和麻风反应的发展情况（逆向反应和麻风结节性红斑反应），以及是否有阳性或阴性杆菌指数，对 753 例患者进行分类。

结果：55.5%的患者报告麻风反应，其中 18.3%（138/753）有逆向反应，5.4%（41/753）出现麻风结节性红斑。麻风反应在诊断后第一年更为活跃，见于 27%（205/753）的患者，而后续随访中仅发现 19%（142/753）的患者有麻风反应。进一步观察统一多药治疗（U-MDT）和常规多药治疗（R-MDT）的少菌型和多菌型麻风病患者（*P*=0.43 和 *P*=0.61）中麻风反应和其他临床表现情况，发现具有高杆菌指数的多菌型患者明显更易发生麻风反应，且更易出现逆向反应。在基线水平，杆菌指数阳性患者尤其是麻风结节性红斑的患者 ML 明窗实验阳性率最高，其次是有神经炎和逆向反应的患者。而没有反应的患者 ML 明窗实验阳性率达到 81.9%，与有反应的患者间的差异无统计学意义（*P*=0.45）。

结论：接受 R-MDT 和 U-MDT 的多菌型和少菌型患者的逆向反应和其他临床表现类似。ML 明窗实验阳性与多菌型麻风病和杆菌指数阳性率相关。但是基线水平的 ML 明窗实验结果在预测麻风反应时的敏感度和特异度有限。

Translated from English version into Chinese by Jian-Hai Yin, edited by Pin Yang

CHINESE CENTER FOR DISEASE CONTROL AND PREVENTION
NATIONAL INSTITUTE OF PARASITIC DISEASES

Les résultats initiaux du test ML Flow permettent-ils de prédire les réactions de la lèpre ? Un examen mené auprès d'une cohorte de patients inclus à l'Essai clinique de polychimiothérapie uniforme pour les patients atteints de lèpre au Brésil

Emerith Mayra Hungria, Regiane Morillas Oliveira, Gerson Oliveira Penna, Lúcio Cartaxo Aderaldo, Maria Araci de Andrade Pontes, Rossilene Cruz, Heitor de Sá Gonçalves, Maria Lúcia Fernandes Penna, Ligia Regina Franco Sansigolo Kerr, Mariane Martins de Araújo Stefani, Samira Bührer-Sékula

Résumé

Contexte : la valeur prédictive de la sérologie dans la détection de l'IgM dirigée contre le glycolipide phénolique/PGL-I dérivé de la bactérie *Mycobacterium leprae* pour identifier les

patients atteints de lèpre étant fortement exposés au risque de développement de réactions reste controversée. La possibilité pour les résultats initiaux du test ML Flow de prédire les réactions de la lèpre a été examinée au sein d'une cohorte de patients inclus à l'Essai clinique de polychimiothérapie uniforme pour les patients atteints de lèpre au Brésil (U-MDT/CT-BR).

Méthodes : il s'agissait d'une étude descriptive concentrée sur les principales manifestations cliniques de patients atteints de lèpre inclus à l'essai U-MDT/CT-BR de mars 2007 à février 2012 auprès de deux centres de traitement de la lèpre de référence au Brésil. À des fins de recherche, 753 patients atteints de la lèpre ont été catégorisés conformément à une classification modifiée de Ridley-Jopling (R&J), sur la base du développement de réactions de la lèpre (réaction reverse/RR et érythème noueux lépreux/ENL) et en fonction de leur obtention d'un indice bacillaire (IB) positif ou négatif.

Résultats : plus de la moitié des patients (55,5 %) ont fait état d'une réaction de la lèpre : 18,3 % (138/753) présentaient une RR et 5,4 % (41/753) affichaient un ENL. L'apparition de réactions de la lèpre était plus fréquente au cours de la première année suivant le diagnostic, comme nous l'avons constaté chez 27 % (205/753) des patients, tandis que 19 % (142/753) avaient développé des réactions au cours de la période de suivi consécutive. Des fréquences similaires des réactions de la lèpre et d'autres manifestations cliniques ont été observées chez les patients atteints de la lèpre paucibacillaire (PB) et multibacillaire (MB) traités dans le cadre d'un U-MDT et d'un MDT standard (R-MDT) (respectivement P =0,43 et P =0,61). Par rapport aux patients atteints d'une lèpre PB, les réactions de la lèpre étaient significativement plus fréquentes chez les patients atteints d'une lèpre MB affichant un IB élevé et plus de patients ont développé une RR qu'un ENL. Néanmoins, la RR et la névrite ont aussi été signalées chez des patients présentant un IB négatif. Au début de l'essai, le taux de résultats positifs au test ML Flow le plus élevé a été observé chez des patients présentant un IB positif, notamment ceux ayant développé un ENL, suivi des patients atteints d'une névrite et d'une RR. Parmi les patients ne présentant aucune réaction, 81,9 % affichaient un résultat positif du test ML Flow. Néanmoins, les différences ne présentaient aucune importance significative par rapport aux patients présentant une réaction (P =0,45).

Conclusions : les patients atteints d'une lèpre MB et PB traités dans le cadre d'un R-MDT et d'un U-MDT ont présenté des fréquences similaires de RR et d'autres manifestations cliniques. Les tests ML Flow positifs étaient associés à une lèpre MB et à des résultats positifs à l'IB. Cependant, les résultats initiaux du test ML Flow ont démontré une sensibilité et une spécificité limitées pour la prédiction du développement de réactions de la lèpre.

Translated from English version into French by eric ragu, through

TRANSLATORS WITHOUT BORDERS

Базовая линия результатов ML Flow теста может ли предсказать реакцию проказы? Расследование среди пациентов, которые участвовали в клинической пробе для единообразной терапии с помощью множественных лекарств для больных проказой в Бразилии

Emerith Mayra Hungria, Regiane Morillas Oliveira, Gerson Oliveira Penna, Lúcio Cartaxo Aderaldo, Maria Araci de Andrade Pontes, Rossilene Cruz, Heitor de Sá Gonçalves, Maria Lúcia

Fernandes Penna, Ligia Regina Franco Sansigolo Kerr, Mariane Martins de Araújo Stefani, Samira Bührer-Sékula

Резюме

Фоновая информация: Предсказательная ценность серологии для обнаружения IgM против палочки Хансена, происходящих из фенольных гликолипид и различения прокажённых, которые находятся в чрезвычайно опасном состоянии, -- всё ещё обсуждаемый вопрос. Могут ли результаты ML Flow теста предсказать реакцию болезни Хансена, этот вопрос исследуется среди больных, которые участвуют в клинической пробе для единообразной терапии с помощью множественных лекарств для больных проказой в Бразилии (U-MDT/CT-BR).

Методы: Данное описательное исследование, концентрируемое на клиническом проявлении пациентов, которые участвовали в U-MDT/CT-BR от марта 2007 до февраля 2012 в двух исслoвательных организциях проказы в Бразилии. Для исследования 753 больных проказой группировали в соответствии с Ridley-Jopling (R&J) классификацией и с развитием реакцией проказы (реверсия реакции/RR и узловатая лепрозная эритема/ENL), несмотря на то, имели они позитивный или негативный бациллярный индекс/BI.Методы:

Результаты: Больше половины пациентов (55.5%) сообщили реакцию проказы: 18.3% имело (138/753) RR и 5.4% (41/753) имело ENL. Частотность реакции проказы в первый год выше после диагноза, около 27% (205/753) из всех пациентов. Однако 19% (142/753) из всех проявило реакцию проказы в следующий период. Подобная частотность реакции проказы и другие клинические проявления наметилось среди олигобациллярных (PB) и полибактериальных (MB) больных проказой, которых лечили U-MDT и регулярный MDT (R-MDT) (P =0.43 и P =0.61, соответственно). В сравнении с PB пациентами, реакция проказы гораздо чаще проявляла среди MB пациентов с высокой BI, и больше пациентов показывало RR, чем ENL. Тем не менее, RR и неврит тоже появились среди пациентов с негативной BI. В базовой линии высокий уровень ML Flow позитивности заметили среди пациентов с позитивной BI, особенно среди тех, кто показал ENL, который следует за пациенами, у которого были нефрит и RR. Среди пациентов бес реакции проказы, 81.9% из них имело позитивный ML Flow, однако, по статистикам разница между пациентами с реакцией и бес реакции была небольшой (P =0.45).

Выводы: Пациенты MB и PB, которые приняли R-MDT и U-MDT, показывают похожую частотность и другие клинические проявления. Позитивный ML Flow тесты связывают с MB проказой and BI позитивностью. Однако, результаты Flow теста показал органиченную чувствительность и специфичность для предсказания развития реакции проказы.

Translated from English version into Russian by Liang Ma

¿Pueden los resultados de línea de base del test de flujo ML lateral predecir las reacciones leprosas? Una investigación en un grupo de pacientes registrados para el Ensayo Clínico de Terapia Multidroga Uniforme para Pacientes con Lepra en Brasil.

Emerith Mayra Hungria, Regiane Morillas Oliveira, Gerson Oliveira Penna, Lúcio Cartaxo Aderaldo, Maria Araci de Andrade Pontes, Rossilene Cruz, Heitor de Sá Gonçalves, Maria Lúcia Fernandes Penna, Ligia Regina Franco Sansigolo Kerr, Mariane Martins de Araújo Stefani, Samira Bührer-Sékula

Resumen

Antecedentes: El valor predictivo de la serología para la detección de la IgM contra el glicolípido fenólico-I/PGL-1 del *Mycobacterium leprae* para identificar pacientes con lepra que puedan correr mayor riesgo de desarrollar reacciones, continúa siendo controversial. Se investigó si los resultados de línea de base del test de flujo ML lateral pueden predecir las reacciones leprosas en un grupo de pacientes registrados en el Ensayo Clínico para la Terapia Multidroga Uniforme para Pacientes con Lepra en Brasil (U-MDT/CT-BR).

Métodos: Este fue un estudio descriptivo que se basaba en las principales manifestaciones clínicas de los pacientes con lepra registrados en el U-MDT/CT-BR entre marzo del 2007 y febrero del 2012 en dos centros de referencia para la lepra en Brasil. A los efectos de la investigación, se categorizaron 753 pacientes con lepra de acuerdo a la clasificación modificada de Ridley-Jopling (R&J) y de acuerdo al desarrollo de reacciones leprosas (reacción reversa/RR y eritema nodoso leproso/ENL), y si habían tenido un índice bacilar /IB positivo o negativo.

Resultados: Más de la mitad de los pacientes (55,5%) reportaron reacción leprosa: 18,3% (138/753) tuvieron una RR y 5,4% (41/753) tuvieron ENL. Las reacciones leprosas fueron más frecuentes en el primer año luego del diagnóstico, como se observó en 27% (205/753) de los pacientes, mientras que 19% (142/753) desarrollaron reacciones durante el seguimiento posterior. Se observaron frecuencias similares de reacciones leprosas y otras manifestaciones clínicas en pacientes leprosos paucibacilares (PB) y multibacilares (MB) tratados con U-MDT y MDT (R-MDT) normal (P =0,43 y P =0,61, respectivamente). Comparado con los pacientes PB, las reacciones leprosas fueron mucho más frecuentes en los pacientes MB con un índice bacilar elevado, y los pacientes desarrollaron más RR que ENL. Sin embargo, también se reportó RR y neuritis en pacientes con IB negativo. En la línea de base, el índice más elevado de positividad de flujo ML se observó en pacientes con IB positivo, en especial aquellos que desarrollaron ENL, seguido de los pacientes que tuvieron neuritis y RR. Entre los pacientes sin reacción, 81,9% fueron positivos al flujo ML. No obstante, las diferencias no fueron estadísticamente significativas cuando se las comparaba con los pacientes con reacción (P =0,45).

Conclusiones: Los pacientes MB y PB tratados con R-MDT y U-MDT mostraron frecuencias similares de RR y otras manifestaciones clínicas. Los tests de Flujo ML se vieron asociados con lepra MB y positividad del IB. Sin embargo, los resultados del test de Flujo ML en la línea de base mostraron sensibilidad y especificidad limitadas para predecir el desarrollo de reacciones leprosas.

Translated from English version into Spanish by Maria Alejandra Aguada, through
