# Supplementary material for: Can baseline ML Flow test results predict leprosy reactions? An investigation in a cohort of patients enrolled in the uniform multidrug therapy clinical trial for leprosy patients in Brazil
Source: Infect Dis Poverty. 2016 Dec 6;5:110. doi: 10.1186/s40249-016-0203-0 (PMC5139020; doi:10.1186/s40249-016-0203-0)
Supplement: Additional file 2: Table S1. — Positivity to ML Flow test according to bacillary index (BI) and type of reaction. (DOC 43 kb) [file 40249_2016_203_MOESM2_ESM.doc]

**SUPPLEMENTARY**

**Table 1. Positivity to ML Flow test according to bacillary index (BI) and type of reaction.**

|  | **ML Flow** | **BI** | N | % |
| --- | --- | --- | --- | --- |
|  |  |  |  |  |
| **RR** | POS | POS | 111 | 67 |
|  | NEG | POS | 20 | 12 |
|  | POS | NEG | 16 | 9,6 |
|  | NEG | NEG | 19 | 11,4 |
| Total |  |  | 166 | 100 |
|  |  |  |  |  |
| **ENL** | POS | POS | 44 | 92 |
|  | NEG | POS | 4 | 8 |
|  | POS | NEG | 0 | 0 |
|  | NEG | NEG | 0 | 0 |
| Total |  |  | 48 | 100 |
|  |  |  |  |  |
| **NEURITIS** | POS | POS | 81 | 54,7 |
|  | NEG | POS | 13 | 8,8 |
|  | POS | NEG | 23 | 15,5 |
|  | NEG | NEG | 31 | 21 |
| Total |  |  | 148 | 100 |
|  |  |  |  |  |
| **OTHER** | POS | POS | 44 | 80 |
|  | NEG | POS | 4 | 7,3 |
|  | POS | NEG | 3 | 5,4 |
|  | NEG | NEG | 4 | 7,3 |
| Total |  |  | 55 | 100 |
|  |  |  |  |  |
| **REACTION** | POS | POS | 86 | 25,7 |
| **FREE** | NEG | POS | 19 | 5,7 |
|  | POS | NEG | 52 | 15,6 |
|  | NEG | NEG | 177 | 53 |
| Total |  |  | 334 | 100 |
