# Supplementary material for: Can baseline ML Flow test results predict leprosy reactions? An investigation in a cohort of patients enrolled in the uniform multidrug therapy clinical trial for leprosy patients in Brazil
Source: Infect Dis Poverty. 2016 Dec 6;5:110. doi: 10.1186/s40249-016-0203-0 (PMC5139020; doi:10.1186/s40249-016-0203-0)
Supplement: Additional file 3: Figure S1. — Receiver Operating Curves (ROC) for ML Flow test in leprosy patients who developed RR, ENL and reaction-free. (DOC 715 kb) [file 40249_2016_203_MOESM3_ESM.doc]

Figure 1. **Receiver Operating Curves (ROC) for ML Flow test in leprosy patients who developed RR, ENL and reaction-free.**

1. **Comparison of ML Flow test results of RR and Reaction-free BI Negative** patients

| **Score** | **Sensitivity%** | **95% CI** | **Specificity%** | **95% CI** |
| --- | --- | --- | --- | --- |
| **≥1** | 45,71 | 28,83% to 63,35% | 76,52 | 70,50% to 81,84% |
| **≥ 2** | 28,57 | 14,64% to 46,30% | 90,00 | 85,37% to 93,55% |
| **≥ 3** | 8,571 | 1,804% to 23,06% | 94,78 | 91,06% to 97,28% |
| **=4** | 8,571 | 1,804% to 23,06% | 99,13 | 96,89% to 99,89% |

| **Area under the ROC curve** |  |
| --- | --- |
| **Area** | 0,6206 |
| **Std. Error** | 0,05485 |
| **95% confidence interval** | 0,5130 to 0,7281 |
| **P value** | 0,02163 |
| **Data** |  |
| **Controls (Reaction-free)** | 230 |
| **Patients (RR)** | 35 |
| **Missing Controls** | 0 |
| **Missing Patients** | 0 |

1. **Comparison of ML Flow test results of RR and Reaction-free** BI Positive patients

| **Score** | **Sensitivity%** | **95% CI** | **Specificity%** | **95% CI** |
| --- | --- | --- | --- | --- |
| **0** | 15,27 | 9,582% to 22,59% | 81,90 | 73,19% to 88,74% |
| **≤1** | 28,24 | 20,73% to 36,77% | 72,38 | 62,80% to 80,66% |
| **≤2** | 45,04 | 36,34% to 53,97% | 53,33 | 43,34% to 63,13% |
| **≤3** | 70,99 | 62,42% to 78,59% | 32,38 | 23,57% to 42,21% |

| **Area under the ROC curve** |  |
| --- | --- |
| **Area** | 0,5027 |
| **Std. Error** | 0,03808 |
| **95% confidence interval** | 0,4281 to 0,5774 |
| **P value** | 0,9426 |
| **Data** |  |
| **Controls (Reaction-free)** | 105 |
| **Patients (RR)** | 131 |
| **Missing Controls** | 0 |
| **Missing Patients** | 0 |

1. **Comparison of ML Flow test results of ENL** and Reaction-free BI Positive patients

| **Score** | **Sensitivity%** | **95% CI** | **Specificity%** | **95% CI** |
| --- | --- | --- | --- | --- |
| **> 1** | 91,67 | 80,02% to 97,68% | 18,10 | 11,26% to 26,81% |
| **> 2** | 72,92 | 58,15% to 84,72% | 27,62 | 19,34% to 37,20% |
| **> 3** | 66,67 | 51,59% to 79,60% | 46,67 | 36,87% to 56,66% |
| **= 4** | 41,67 | 27,61% to 56,79% | 67,62 | 57,79% to 76,43% |

| **Area under the ROC curve** |  |
| --- | --- |
| **Area** | 0,5657 |
| **Std. Error** | 0,04964 |
| **95% confidence interval** | 0,4684 to 0,6630 |
| **P value** | 0,1931 |
| **Data** |  |
| **Controls (Reaction-free)** | 105 |
| **Patients (ENL)** | 48 |
| **Missing Controls** | 0 |
| **Missing Patients** | 0 |
